# The multikinase inhibitor axitinib in the treatment of advanced hepatocellular carcinoma: the current clinical applications and the molecular mechanisms

**DOI:** 10.3389/fimmu.2023.1163967

**Published:** 2023-05-31

**Authors:** Hao Jiang, Jian Liao, Liezhi Wang, Chong Jin, Jinggang Mo, Sheng Xiang

**Affiliations:** ^1^ Department of General Surgery, Taizhou Central Hospital (Taizhou University Hospital), Taizhou, Zhejiang, China; ^2^ Department of Nephrology, Jiaxing Hospital of Traditional Chinese Medicine, Jiaxing, Zhejiang, China; ^3^ Department of General Surgery, Tiantai People’s Hospital, Taizhou, Zhejiang, China

**Keywords:** axitinib, hepatocellular carcinoma, survival, mechanism, tyrosine kinase

## Abstract

Advanced hepatocellular carcinoma (HCC) is a formidable public health problem with limited curable treatment options. Axitinib, an oral tyrosine kinase inhibitor, is a potent and selective second-generation inhibitor of vascular endothelial growth factor receptor (VEGFR) 1, 2, and 3. This anti-angiogenic drug was found to have promising activity in various solid tumors, including advanced HCC. At present, however, there is no relevant review article that summarizes the exact roles of axitinib in advanced HCC. In this review, 24 eligible studies (seven studies in the ClinicalTrials, eight experimental studies, and nine clinical trials) were included for further evaluation. The included randomized or single-arm phase II trials indicated that axitinib could not prolong the overall survival compared to the placebo for the treatment of advanced HCC, but improvements in progression free survival and time to tumor progression were observed. Experimental studies showed that the biochemical effects of axitinib in HCC might be regulated by its associated genes and affected signaling cascades (e.g. VEGFR2/PAK1, CYP1A2, CaMKII/ERK, Akt/mTor, and miR-509-3p/PDGFRA). FDA approved sorafenib combined with nivolumab (an inhibitor of PD-1/PD-L1) as the first line regimen for the treatment of advanced HCC. Since both axitinib and sorafenib are tyrosine kinase inhibitors as well as the VEGFR inhibitors, axitinib combined with anti-PDL-1/PD-1 antibodies may also exhibit tremendous potential in anti-tumoral effects for advanced HCC. The present review highlights the current clinical applications and the molecular mechanisms of axitinib in advanced HCC. To move toward clinical applications by combining axitinib and other treatments in advanced HCC, more studies are still warranted in the near future.

## Background

Liver cancer, a hypervascular tumor, ranks as the 6th most common malignancy worldwide (1). Besides, it is the third most common cause of cancer-associated mortalities (1). According to the Cancer statistics 2023 on the category of liver cancer, it is predicted to have 41,210 new cases and 29,380 deaths in the United States (2). Hepatocellular carcinoma (HCC) is the most common form of primary liver cancer, comprising over 80% of cases. HCC is prevalent in Eastern Asia and Africa, where mortality and incidence are highest (3). In patients with distant metastases of HCC, the 5-year survival rate is only 2.4%. There are several factors that contribute to the development of HCC, including viral infections (the main cause), alcohol-related liver cirrhosis, genetic mutations, diabetes mellitus, obesity, smoking, exposure to chemical carcinogens, and non-alcoholic steatohepatitis, etc (4, 5). It frequently occurs in patients with chronic liver disease. Several factors play an important role in the pathophysiology of HCC, these include but are not limited to genetic predisposition, cellular microenvironment, and immune cells (6).

For early-stage HCC patients, resection, transplantation, and local ablation are the preferred treatments. In patients with intermediate stages of the disease, transarterial chemoembolization (TACE), local ablation treatments (i.e., radiofrequency ablation), and radiotherapy (RT) techniques are recommended, while those with advanced disease should first receive systemic treatments (5). Multiple randomized studies demonstrated that a better survival rate was observed in patients treated with TACE versus those treated only symptomatically (7). In the case of advanced or unresectable HCC, systemic therapies are an effective treatment modality. Specifically, sorafenib is a first-line systemic treatment for unresectable HCC (8). In recent years, immunotherapy (i.e., ramucirumab, nivolumab, and pembrolizumab) also play an important role in treating patients with advanced HCC who failed the treatment of sorafenib (9).

Axitinib is a selective tyrosine kinase inhibitor (TKI) of vascular endothelial growth factor receptors (VEGFRs) 1, 2, and 3 (10). This anti-angiogenic drug was found to have promising activity in various solid tumors. Axitinib has been approved for the treatment of advanced renal cell carcinoma (RCC) after prior systemic therapies have failed (11). According to mounting evidence, angiogenesis is confirmed to contribute to the pathogenesis and progression of HCC *via* several signal pathways, e.g. VEGF/VEGF receptor (VEGFR) signalling (12). Since both HCC and RCC are hypervascular cancers that can be effectively controlled by angiogenesis inhibitors, axitinib has been studied as a second-line treatment option in advanced HCC after sorafenib failed (13).

At present, numerous phase I/II clinical trials on axitinib have been completed and some of them have been published. Nevertheless, there is still no relevant review for summarizing all the clinical and experimental evidence of the effects of axitinib on advanced-stage HCC. Therefore, we conducted this study, which might better illustrate the status of axitinib in treating advanced HCC.

## Overview of axitinib

Axitinib, known as AG 013736 (Inlyta; Pfizer Inc, New York, New York), an oral tyrosine kinase inhibitor, is a potent and selective second-generation inhibitor of VEGFR1, 2, and 3 (14). As engagement of these VEGFRs, cell growth and angiogenesis are stimulated, leading to tumor growth. The VEGF/VEGFR pathway plays an essential role in normal vascular development. Besides, it is also associated with the survival, growth and metastasis of tumors (15). Axitinib is a multitarget tyrosine kinase inhibitor that can not only suppress EGFR1/2/3 and the gene cKIT, but also the platelet-derived growth factor receptor (PDGFR) (16). Axitinib is an indazole derivative synthesized by chemical synthesis with a molecular weight of 386,47 Da (17). It can bind to the inactive conformation of the catalytic domain of VEGF receptor tyrosine kinases (RTKs). Taking axitinib orally produces rapid absorption and a maximum plasma concentration within four hours. When taken at therapeutic doses, axitinib has a high protein binding rate exceeding 99%, preferring albumin over other proteins. Metabolism of axitinib occurs predominantly in the liver by CYP3A4/5, but to a lesser extent by CYP1A2, 2C19, and UGT1A1, producing pharmacologically inactive metabolites (17). The majority of axitinib is excreted in the feces as a result of hepatobiliary excretion, while less than 20% is excreted by the kidney. The plasma concentration of axitinib increased significantly in patients with moderate hepatic impairment (Child-Pugh B), but not in patients with mild impairment (Child-Pugh A) (17, 18).

One of the on-target effects of VEGFR-tyrosine kinase inhibitors include an increase in blood pressure. As a result, an increase in diastolic blood pressure > 90 mm Hg was associated with axitinib’s effectiveness in solid tumors (19). Higher exposure and diastolic blood pressure were found to be independently correlated with longer PFS as wells as OS and a higher probability of partial response (20).

A starting dose of 5 mg twice daily of axitinib is recommended with continuous daily administration, while dose adjustments are recommended according to individual tolerability. It is recommended to raise the dose to 7 mg after two weeks in patients who have shown good tolerance (e.g. no adverse events > grade 2, no increases in BP > 150/90 mm Hg or introduction of antihypertensive treatment) (17). If adverse events occur, a dose reduction could be necessary followed by reintroduction of treatment.

Axitinib was approved by both American and European agencies in 2012 for the treatment of advanced renal cell carcinoma following one prior systemic therapy failure (21). VEGF inhibitors (e.g., bevacizumab) and VEGFR inhibitors (e.g., sorafenib, sunitinib, pazopanib, and axitinib) are effective against advanced renal cell carcinoma. They are approval for treating advanced RCC either as monotherapy or in combination with interferon-alpha (bevacizumab) (14). In addition to kidney cancer, axitinib was also found to improve the outcomes in patients with other malignancies, included head and neck tumors, thyroid cancer, breast cancer, non-small-cell lung cancer, pancreatic cancer, melanoma, and HCC (16, 22, 23).

## Relevant studies reporting the roles of axitinib in HCC

To identify the eligible studies, we searched several electronic databases, including MEDLINE (PubMed), the Cochrane Library databases, EMBASE (OVID), PsychINFO, SCOPUS, and ISI, from their inception until December 31, 2022. Among the studies included, only those studies reported using English were considered to be eligible. PubMed search keywords with various combinations were as follows: (((((“Axitinib”[Mesh]) OR (AG 013736)) OR (AG013736)) OR (AG-013736)) OR (Inlyta)) AND (((((((((((((((((((“Carcinoma, Hepatocellular”[Mesh]) OR (Carcinomas, Hepatocellular)) OR (Hepatocellular Carcinomas)) OR (Liver Cell Carcinoma, Adult)) OR (Liver Cancer, Adult)) OR (Adult Liver Cancer)) OR (Adult Liver Cancers)) OR (Cancer, Adult Liver)) OR (Cancers, Adult Liver)) OR (Liver Cancers, Adult)) OR (Liver Cell Carcinoma)) OR (Carcinoma, Liver Cell)) OR (Carcinomas, Liver Cell)) OR (Cell Carcinoma, Liver)) OR (Cell Carcinomas, Liver)) OR (Liver Cell Carcinomas)) OR (Hepatocellular Carcinoma)) OR (Hepatoma)) OR (Hepatomas)). In addition, registered studies in the ClinicalTrials (https://clinicaltrials.gov/) were also included for data analyzing.

## Studies in the ClinicalTrials

As listed in the page in ClinicalTrials.gov, seven clinical studies have been registered focusing on the safety and the efficacy of Axitinib in treating advanced HCC (**Table 1**). Six out seven (86%) of these trials have been completed, while one trial was withdrawn. All these studies were either Phase I or Phase II trial. The areas that these trials conducted included Canada, Hong Kong, Chinese Taipei, and Multi-center in multiple countries. The sample size in these studies ranged from 9 to 224 participants. The age among these participants were >18 years. The dosage regimens for Axitinib administration were 5 mg twice daily orally. The treatment of Axitinib continued until progressive disease, intolerable toxicity, or patient withdrawal. The treatment cycle included 4 weeks, 8 weeks, and 3-6 months. The combination therapies with Axitinib, including TACE, radiotherapy, Avelumab, and Crizotinib. The responsible party included Pfizer, National Taiwan University Hospital, Chinese University of Hong Kong, and Shin Kong Wu Ho-Su Memorial Hospital. In the study of NCT01210495 (Phase 2), the outcomes of the clinical trial were non-significant OS [12.7 (10.2 to 14.9) for Axitinib vs 9.7 (5.9 to 11.8) for Placebo, HR=0.907, 95%CI: 0.646-1.274, *P*=0.2872] but a significant median PFS [3.6 (2.3 to 4.6) for Axitinib vs 1.9 (1.9 to 3.5) for Placebo, HR=0.618, 95%CI: 0.438-0.871, *P*=0.0039] as well as a significant Time to Disease Progression (TTP) (HR=0.621, 95%CI: 0.434-0.889, *P*=0.006). In another study of NCT03289533 (Phase 1), the investigators demonstrated that patients received avelumab 10 mg/kg Q2W in combination with axitinib 5 mg BID turned out to be a TTP of 5.52 months (1.91 to 7.39), an objective response of 13.6% (2.9 to 34.9), a disease control of 68.2% (45.1 to 86.1), and a Time to Tumor Response (TTR) of 1.91 (1.9 to 3.7). Five out of seven studies did post the outcomes. Serious adverse events were reported at 46.6% [Axitinib: 62/133 (46.6%) vs Placebo: 16/68 (23.5%)] and36.36% (8/22).

**Table 1:** Studies registered in the ClinicalTrials.

Clinical Trials ID	Study area	Status	Cancer type	Number of patients	Age (years)	Therapies (Axitinib)	Time Frame	Responsible Party	Outcomes	Serious Adverse Events (%)
NCT01334112, Phase 2	Canada	Completed	Advanced HCC	30	Over 18	5 mg, BID, Orally, in cycles of 4 weeks	January 2011 to March 2018	University Health Network, Toronto; Pfizer	No Results Posted	No Results Posted
NCT01273662, Phase 2	Multi-center	Completed	Advanced HCC	45	Over 18	5 mg, BID, Orally	April 2011 to December 2016	National Taiwan University Hospital	No Results Posted	No Results Posted
NCT01210495, Phase 2	70 centers (13 countries)	Completed	Advanced HCC	224	Over 18	1 mg, 5 mg BID, Orally vs Placebo; Duration: 3-6 months	December, 2010 to December, 2016	Pfizer	Median OS: 12.7 (10.2 to 14.9) for Axitinib vs 9.7 (5.9 to 11.8) for Placebo, HR=0.907, 95%CI: 0.646-1.274, P=0.2872; Median PFS: 3.6 (2.3 to 4.6) for Axitinib vs 1.9 (1.9 to 3.5) for Placebo, HR=0.618, 95%CI: 0.438-0.871, P=0.0039; TTP: HR=0.621, 95%CI: 0.434-0.889, P=0.006	Axitinib: 62/133 (46.6%) vs Placebo: 16/68 (23.5%)
NCT01352728, Phase 2	Hong Kong	Completed	Unresectable HCC	50	Over 18	TACE+ 5 mg Axitinib, Daily, Orally	May, 2011 to June, 2018	Chinese University of Hong Kong	No Results Posted	No Results Posted
NCT02814461, Phase 1	Chinese Taipei	Completed	Advanced HCC	9	20-85	1mg, 2mg, 3mg, BID, Orally; Combination with radiotherapy; Duration: total 8 weeks	June, 2016 to November 2018	Shin Kong Wu Ho-Su Memorial Hospital	No Results Posted	No Results Posted
NCT03289533, Phase 1	NA	Completed	Advanced HCC	22	Over 20	avelumab 10 mg/kg Q2W in combination with axitinib 5 mg BID	September, 2017 to September, 2020	Pfizer	TTP=5.52 (1.91 to 7.39); Objective Response= 13.6 (2.9 to 34.9); Disease Control= 68.2 (45.1 to 86.1) TTR= 1.91 (1.9 to 3.7)	8/22 (36.36%)
NCT01441388, Phase 1	NA	Withdrawn	Advanced HCC, Glioblastoma	NA	Over 18	Crizotinib plus axitinib	September 27, 2011 to December 20, 2011	Pfizer	No Results Posted	No Results Posted

NA, Not available; HCC, Hepatocellular carcinoma; HR, Hazard ratio; CI, Confidence interval; OS, Overall Survival; PFS, Progress Free Survival. TACE, Transarterial Chemoembolisation; TTP, Time to Disease Progression; TTR, Time to Tumor Response.

## Experimental (preclinical) studies

At present, there were eight experimental studies had reported the molecular biological effects of axitinib in HCC development (**Table 2**). These included studies were conducted in the USA, China, Australia, Canada, and Singapore. The cell lines used in these studies included various types of HCC cells. It has been shown that axitinib inhibits cellular phosphorylation of VEGFR-2, VEGF-induced endothelial cell survival, tube formation, and vascular permeability in preclinical studies (24). A significant delay in the growth of human xenograft tumors was observed when VEGFR-2 was expressed, suggesting a therapeutic potential for this protein (18, 24). Therefore, inhibition of VEGFR-2, like axitinib, might be effective for treating multi-blood vessel solid tumor (e.g., HCC). In an early preclinical study developed by Ma et al. (25), the author found that axitinib modulated the anti-tumor activity of metronomic cyclophosphamide in multiple ways. Axitinib caused a rapid decrease of blood vessel perfusion, exhibiting a transient pro-apoptotic activity on the cancer cells.

**Table 2:** Experimental (preclinical) studies reported that biological effects of axitinib in HCC.

Study	Study area	Materials	Signaling pathway	Molecular mechanisms
Chen et al., 2013	China	Cell lines and human hepatoma tissues	VEGFR2/PAK1	Axitinib is an efficient inhibitor for Klotho-mediated anoikis resistance, which might provide a therapeutic intervention for those HCC patients with high Klotho expression.
Gu et al., 2013	Australia	Human liver tissue	CYP1A2	Axitinib potently suppressed CYP1A2-dependent 7-ethoxyresorufin O-deethylation activity at a low level.
Liu et al., 2015	China	Human HCC cell line	ERK, VEGFR	5 µM Axitinib significantly inhibited cancer cell proliferation and viability; Insufficient radiofrequency ablation enhanced liver cancer cell proliferation by activating CaMKII/ERK-dependent VEGF overexpression.
Lv et al., 2016	China	liver tumor –bearing rabbits	NA	Axitinib exhibits antitumor efficacy on liver tumor; tumor growth was significantly suppressed by Axitinib compared with the control, tended to be smaller with higher dosages.
Chiew et al., 2017	Singapore	HCC cell line, HUVEC, and mouse model	Akt/mTor	Axitinib induces HUVEC apoptosis and reduced vascular networks, but is unable to kill liver cancer cells; Axitinib had a lower anti-angiogenic effect than sunitinib.
Amin et al., 2019	Canada	Cells and clinical samples	Glycolysis and the citric acid cycle	Under axitinib treatment, 5,6-dihydrouracil and glycopyranose increased, while glutamic acid, glutamine, and a lactose derivative decreased with treatment-response.
Lin et al., 2019	China	Rats and cells	NA	Axitinib inhibited the metabolism of loperamide noncompetitively *in vitro* and affect the pharmacokinetic characteristics of loperamide *in vivo*.
Li et al., 2020	China	HCC cells	miR-509-3p/PDGFRA	LINC00467 promoted the proliferation and invasion of the HCC cells. Besides, high level of LINC00467 contributed to Axitinib resistance of HCC through miR-509-3p/PDGFRA axis.

NA, not available.

Klotho is an aging suppressor gene, while its molecular mechanisms in hepatocarcinogenesis remains unclear (26). Chen et al. (27) demonstrated that Axitinib was an efficient VEGFR2 inhibitor for Klotho-mediated anoikis resistance. Axitinib exhibited the anti-tumor function of Klotho in suppressing anoikis resistance and anchorage-independent growth *via* inhibiting VEGFR2/PAK1 signaling, which provide a therapeutic intervention for those HCC patients with high Klotho expression. Targeting Klotho/VEGFR2/PAK1 signaling pathway by Axitinib might be an effective treatment for the hepatoma resistance to anoikis in hepatocarcinogenesis, thus providing an intervention with HCC metastasis (27).

In recent years, tyrosine kinase inhibitors (TKIs) and multikinase inhibitors (MKIs) have gained increasing importance as oncology drugs that improve the treatment of many types of tumors (28), including HCC. MKI axitinib was found to be effective in inhibiting CYP1A2-catalyzed O-deethylation of 7-ethoxyresorufin by cDNA-expressed enzymes and human liver microsomes (29). As a result, co-administering axitinib with alternate substrates of CYP1A2 may result in interactions of each other, together contributed to improving the efficacy of pharmacological treatments.

As an alternative to traditional surgery, radiofrequency ablation (RFA) is widely used for the treatment of HCC (30). Compared with surgical resection, RFA is a simpler modality that inflicts less injury to the liver. Mounting studies have reported that RFA in combination with immunosuppressant could increase the clinical efficacy of HCC. Liu et al. (31) found that insufficient RFA enhanced HCC cancer cell proliferation by activating CaMKII/ERK-dependent VEGF overexpression, while 5 µM Axitinib could significantly suppress HCC cell proliferation and viability *via* inhibiting VEGFR.

During tumor angiogenesis, tumor vessels have structural and functional abnormalities, and they are essential for tumor growth, progression, and metastasis (32). Inhibiting tumor vasculature may be an effective cancer therapy target. Lv et al. (33) established a VX2 liver tumor–bearing rabbit model and further demonstrated that axitinib exhibited antitumor efficacy on HCC animal model. In this study, the authors also found that tumor growth was significantly suppressed by Axitinib compared with the controls. In addition to the above findings, they further suggested that the therapeutic effects of Axitinib in suppressing VX2-mediated HCC in rabbits could be noninvasively and quantitatively monitored with CT spectral imaging parameters.

At present, treatment efficacy is commonly monitored by computed tomography (CT) scans and magnetic resonance imaging (MRI). Treatment effects for solid tumors are typically characterized by the reduction in tumor size or tumor attenuation (34). Amin et al. (35) demonstrated that 5,6-dihydrouracil and glycopyranose increased after treatment with axitinib, while glutamic acid, glutamine, and a lactose derivative decreased with treatment-response. In Amin et al.’s study, the clinical samples were collected at 2-4 weeks after initiation of axitinib. Since such a phenomenon of the treatment-related changes in the metabolome was detected, the clinicians may identify individuals who are not benefiting from a chemotherapeutic agent, which may serve as a part of an adaptive treatment algorithm.

Among axitinib patients, diarrhea is the most common adverse reaction, with a mean frequency in all grades exceeding 50% (36). Loperamide, an opioid receptor agonist, is widely prescribed to treat chronic diarrhea and acute diarrhea as well (37). Chemotherapy-induced diarrhea is commonly treated with high-dose loperamide, which is considered the standard first-line treatment. As a result, concurrent use of loperamide and axitinib may ensure the efficacy of antitumor properties as well as minimize the side effects. However, loperamide is a substrate of CYP2C8, CYP3A4 and P-gp, suggesting there may be a direct correlation between axitinib and loperamide (38). Lin et al. (39) showed that axitinib inhibited the metabolism of loperamide noncompetitively *in vitro* and affected the pharmacokinetic characteristics of loperamide *in vivo*. The peak time of loperamide increased while blood clearance decreased under the impact of axitinib. As a result, since the pharmacokinetics of LOP have been altered remarkably, it is recommended to avoid the combination of axitinib and LOP, even if the two drugs are administered at therapeutic doses.

The PDGFR gene has been reported to be highly expressed in HCC (40). PDGFR can be regulated by Axitinib, while PDGFRA and PDGFRB are two isoforms of PDGFR. High expression of PDGFRA/B was found to be closely associated with low OS in HCC patients (41). A recent study conducted by Li et al. (42) indicated that LINC00467 promoted the proliferation and invasion of the HCC cells. The authors also found that high level of LINC00467 contributed to Axitinib resistance of HCC through miR-509-3p/PDGFRA axis. LINC00467, one of the lncRNAs being detected, has been found to serve as an oncogene in multiple cancers, including neuroblastoma, lung cancer, and colorectal cancer (43). In Li et al.’s study (42), LINC00467 was upregulated in HCC samples as compared to the normal live tissues by analyzing TCGA database. The authors further observed that LINC00467 inhibition might suppress the proliferation and invasion but promoted the apoptosis of the HCC cells. They also suggested that LINC00467 might involve in the Axitinib resistance of HCC.

Inconsistent with the above studies, Chiew et al. (44) reported that Axitinib induced HUVEC apoptosis and reduced vascular networks *via* the Akt/mTOR signaling pathway, but is unable to kill the HCC cells. They concluded that Axitinib had a lower anti-angiogenic effect than sunitinib for treating HCC in a 3D co-culture spheroid of tumor cells.

**Figure 1:**
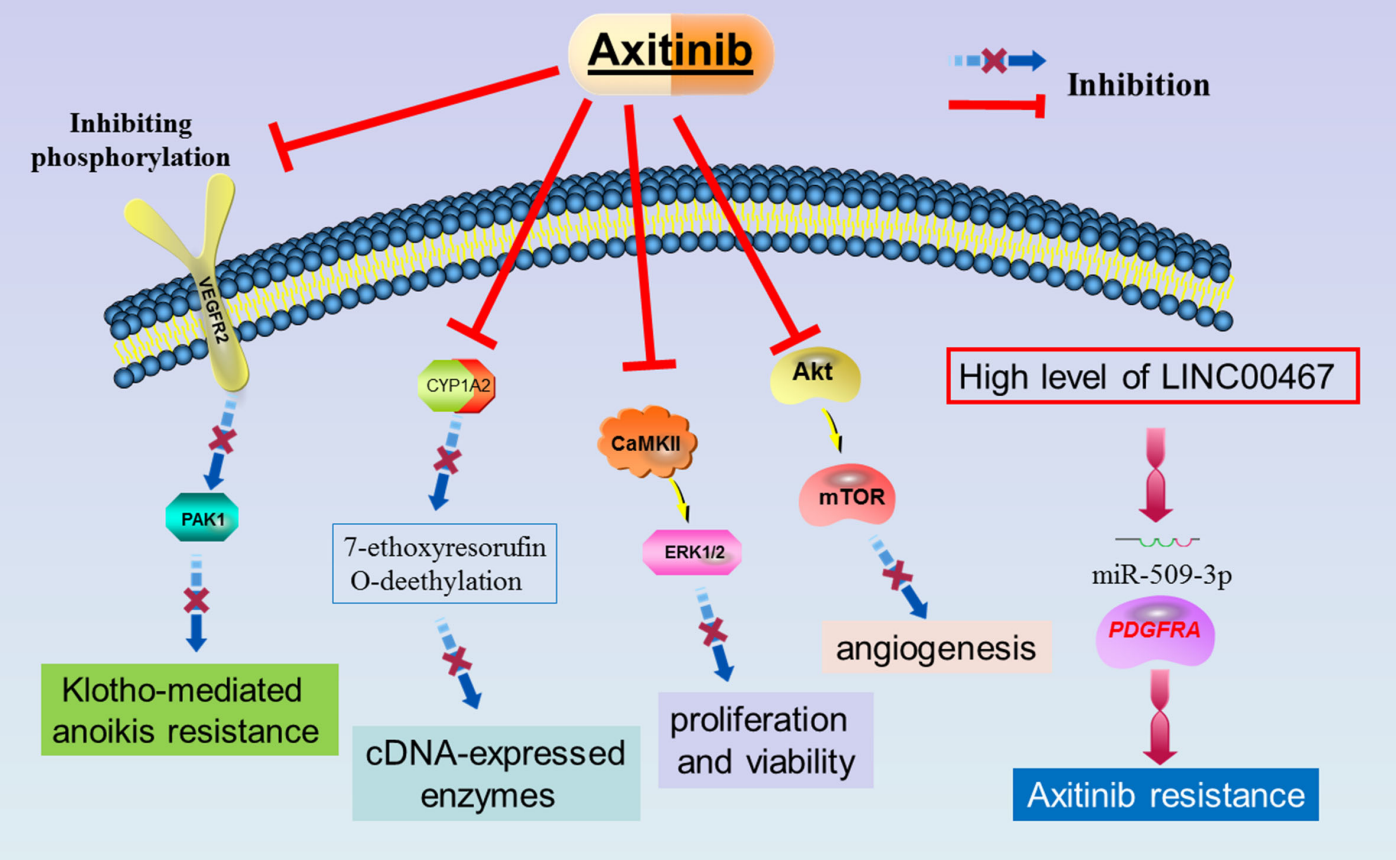
The molecular mechanism underlying the roles of axitinib in advanced HCC.

Taken together, the above experimental/preclinical studies demonstrated that the molecular mechanisms of anti-tumor action of the Axitinib presents itself as a multifaceted process. The associated signaling molecules included VEGFR2/PAK1, CYP1A2, CaMKII/ERK, Akt/mTor, and miR-509-3p/PDGFRA. **Figure 1** showed the molecular mechanism underlying the roles of axitinib in advanced HCC. The biochemical mechanisms might be associated with glycolysis and the citric acid cycle. These *in vitro* and *in vivo* studies may improve the understanding of the biological functioning of Axitinib in the treatment of HCC, indicating that Axitinib is worthy to be popularized in clinical practice.

## Published clinical trials

After a systematically search in the six common databases, we have identified nine published clinical trials (**Table 3**). The year of publication among the included studies ranged from 2015 to 2021. The study location/area/region included Japan, Canada, China, and multi-center involving multiple countries. The study design included phase I, phase II, and case report, either single arm or randomized. The sample size ranged from 1 to 202 participants. The tumor stage of HCC included advanced, metastatic, and inoperable HCC. The age of the participants ranged from 18 to 84. The administration of Axitinib was mainly 5 mg twice daily orally, while 1 mg, 2mg, 3mg, and 7mg twice daily were also investigated. The treatment period included 4 weeks, 8 week, and 16 weeks. The combination therapies included transarterial chemoembolization, radiotherapy, and avelumab intravenously. The most common adverse event reported in the nine included studies was hypertension. Other side-effect included diarrhea, decreased appetite, thrombocytopenia, ulcerative oral mucositis, rash, polyhidrosis, fatigue, emesis, hyperbilirubinemia, high transaminases, abdominal pain, asthenia, palmar-plantarerythrodysesthesia syndrome, proteinuria, alkaline phosphatase, and bilirubin.

**Table 3:** Published studies reported the effect of axitinib in advanced HCC.

Study and references	Study area	Clinical phase	Cancer type, Number of patients	Age (years)	Axitinib	Therapeutic effects or molecular mechanisms	Adverse events (%)
Kang et al., 2015	Japan	II/Randomized	Advanced HCC, 202	25-84	5 mg BID in 4-week cycles, orally	Axitinib did not improve OS over placebo (HR=0.907, 95%CI: 0.646-1.274, median months: 12.7 vs 9.7). But it could significantly improve the PFS, TTP, and BCR.	Diarrhoea (54%), hypertension (54%), decreased appetite (47%).
McNamara et al., 2015	Canada	Single-arm phase II	Advanced HCC, 30	27-85	5 mg twice daily orally, 16 weeks	Tumor control rate at 16 weeks was 42.3% (95% CI: 22.3%-63.1%).	Hypertension (16.7%), thrombocytopenia (13.3%), diarrhea (10.0%)
Zhang et al., 2015	China	Case report	Metastatic HCC, 1	64	7 mg twice daily orally	Combined axitinib and cabozantinib (50 mg, qd, po) after failed sorafenib treatment. The patient had a total survival of 10 months.	Ulcerative oral mucositis, rash, polyhidrosis, fatigue, loss of appetite, emesis, and elevated blood pressure
Lo et al., 2016	Multi-center Clinical Trial	Single arm phase II	Advanced HCC, 15	18-78	5 mg twice daily orally	No significant association was found in the PFS (P=0.310) or progression at 16 wk (P=0.849). But a borderline statistically significant OS was observed (P=0.050).	NA
Chan et al., 2017	China	Single arm phase II (follow-up: 39.9 months)	Inoperable HCC, 50	61.8 ± 7.6	5 mg twice daily orally combined with transarterial chemoembolization	The 2-year OS rate was 43.7%, and the median OS was 18.8 months. The median TTP was 10.4 months (95% CI: 5.4-12.7) and the PFS was 8.4 months (95% CI: 3.9-11.2).	Hyperbilirubinemia (14%), increase in transaminases (44%), and abdominal pain (24%)
Kudo et al., 2018	Multi-center Clinical Trial	II/Randomized	Advanced HCC, 202	65	5 mg twice daily orally in 4-week cycles	Median OS in the axitinib and the placebo arm, respectively, was 12.3 months and 11.2 months in non-Asia (P=0.465) and 13.5 months and 6.3 months in Asia (P=0.226). PFS significantly longer in the axitinib group (3.6 months) than in the placebo arm (1.8 months) in Asia (HR 0.556, 95% CI 0.370–0.835; P= 0.0023) but not in non-Asia group.	Hypertension, diarrhea, asthenia, fatigue, etc.
Lin et al., 2020	China	II/Randomized	Advanced HCC, 45	32-76	5 mg twice daily orally	The disease control rate was 62.2%. Median PFS and OS were 2.2 months and 10.1 months, respectively.	Fatigue (60%), anorexia (57%), hypertension (56%), and rash (49%), etc.
Kudo et al., 2021	Japan	Phase Ib	Advanced HCC, 22	20-84	5 mg twice daily orally combined avelumab 10 mg/kg intravenously every 2 weeks	The objective response rate was 13.6% (95% CI: 2.9–34.9%) per RECIST 1.1 and 31.8% (95% CI: 13.9–54.9%) per mRECIST for HCC.	Hypertension (50.0%), palmar-plantarerythrodysesthesia syndrome (22.7%), and decreased appetite (13.6%).
Yang et al., 2021	China	Phase I	Advanced HCC, 9	37-83	1 mg, 2mg, and 3mg twice daily for 8 weeks in combination with radiotherapy	Overall response rate was 66.7%. 1−year OS was 66.7% and median PFS was 7.4 months.	Hypertension, proteinuria, increased alanine transaminase, alkaline phosphatase, and bilirubin.

NA, Not available; HCC, Hepatocellular carcinoma; HR, Hazard ratio; CI, Confidence interval; OS, Overall Survival; PFS, Progress Free Survival; RECIST, Response Evaluation Criteria in Solid Tumors.

A randomized phase II study developed by Kang et al. (45) in 2015 recruited 134 advanced HCC patients under axitinib treatment (5 mg BID in 4-week cycles, orally) and 68 patients received placebo. The investigators found that Axitinib did not improve OS over placebo (HR=0.907, 95%CI: 0.646-1.274, median months: 12.7 vs 9.7). But Axitinib could significantly improve the PFS, TTP, and BCR (all P<0.05). Interestingly, though the OS between the axitinib and placebo group did not reach statistical significance, the authors found the OS was better in some subgroups, such as Eastern Cooperative Oncology Group performance status (ECOG PS) 1, excluding those intolerant to prior antiangiogenic therapy, as well as those patients with baseline α-fetoprotein ≥400 ng/ml. In regard to PFS, the survival time was significantly better in patients under axitinib treatment at Asian, but not non-Asian. In this study, the authors also investigated several serum soluble protein biomarkers to predict the survival of the patients. The results showed that patients with low baseline serum level of E-selectin or stromal cell-derived factor-1 had a significantly higher OS than those with a high level. In addition, the investigators further found a prognostic association between lower baseline levels of circulating IL-6 or angiopoeitin-2 and longer OS (all P<0.05). The adverse events in this study included diarrhoea (54%), hypertension (54%), and decreased appetite (47%). This study indicated that axitinib could prolong rather the PFS and TTP than the OS of patients with advanced HCC, with an acceptable safety profile. E-selectin, stromal cell-derived factor-1, IL-6, and angiopoeitin-2 were the potential prognostic and predictive biomarkers in the action of axitinib on advanced HCC.

In a previous single-arm phase II study in Canada, McNamara et al. (46) recruited 30 advanced HCC patients and all the patients received Axitinib 5 mg twice daily orally for16 weeks (without a placebo group). The authors found the tumor control rate at 16 weeks was 42.3% (95% CI: 22.3%-63.1%) through the standards of RECIST1.1. The median duration of tumor control on Axitinib treatment was 3.6 months (95% CI, 2.8-9.2 months). PFS (P= 0.0005) and OS (P = 0.04) were found to be associated with the greatest percentage change from baseline in the sum of the diameters of tumor lesions by using RECIST 1.1. Similar trend was found in Choi criteria but not for mRECIST criteria. For different race of the investigated patients, the median OS in Asians was 9.7 months vs 6.6 months for non-Asian (P=0.19), no significant different was found. In this study, no biomarker was identified that could be predicted the PFS and the OS (all P>0.05). The adverse events reported in this trial included hypertension (16.7%), thrombocytopenia (13.3%), and diarrhea (10.0%). In the further analysis, median PFS in patients who developed grades 1-3 hypertension was 10.7 months vs 2.8 months in those who did not (P=0.0004). In aspect of the OS, patients who developed hypertension was 17.2 months vs 6.0 months in those who did not (P < 0.0001). This study suggested that axitinib could encourage tolerable clinical activity in HCC patients, which need more potential biomarkers to evaluate the responses of axitinib treatment.

At present, in addition to McNamara et al.’s study, there were another two single arm phase II studies had published. Lo et al. (13) conducted a multi-center clinical trial which investigated 15 advanced HCC patients in Australia, Canada, and UK. The researchers reported that no significant association was found in the PFS (P=0.310) or progression (P=0.849) after the treatment of axitinib 5 mg twice daily orally at 16 weeks. However, they observed a borderline statistically significant on the OS (P=0.050), even though limited by a small sample size. On the other hand, the authors also indicated that dynamic contrast-enhanced ultrasound (DCE-US) might be potentially useful in monitoring early tumor response of advanced HCC to axitinib treatment. Besides, tumor fractional blood volume measurement using the DCE-US infusion technique might be a promising imaging biomarker to predict OS in patients with advanced HCC who under axitinib treatment. Another single arm phase II trial developed by Chan et al. (47) had recruited 50 inoperable HCC patients who treated with 5 mg Axitinib twice daily orally combined with transarterial chemoembolization with a follow-up of 39.9 months. This study showed that the 2-year OS rate was 43.7% and the median OS was 18.8 months after the treatment of the combination of Axitinib and transarterial chemoembolization. The median TTP was 10.4 months (95% CI: 5.4-12.7) and the PFS was 8.4 months (95% CI: 3.9-11.2). In this study, the common adverse events of grade 3 were increase in transaminases (44%), abdominal pain (24%), and hyperbilirubinemia (14%), which might be associated with the treatment of transarterial chemoembolization. Under the treatment of Axitinib, the side-effect included hypertension (24%) and hand-foot skin reaction (14%). The authors also investigated the predictive parameters of efficacy of the treatment. They found that patients who developed hypertension had a better median PFS when compared to those without a hypertension (11.6 months vs 4.5 months, P= 0.0017). Similarly, the median OS was also better in those with hypertension than the absence of hypertension (25 months vs 14.1 months, P= 0.0222). Interestingly, a higher grade of hypertension also was correlated to a better median PFS (P= 0.004). In the multivariate analyses, the presence and grading of hypertension and Eastern Cooperative Oncology Group (ECOG) performance status were the independent prognostic factors for OS (all P<0.05). The above studies revealed that combining axitinib with TACE or DCE-US might effective for patients with inoperable HCC.

A previous phase II randomized multi-center clinical trial (48) demonstrated that the median OS in the axitinib (5 mg twice daily orally in 4-week cycles) and the placebo arm, respectively, was 12.3 months and 11.2 months in non-Asia (P=0.465) and 13.5 months and 6.3 months in Asia (P=0.226). These results indicated axitinib did not prolong the OS of the advanced HCC patients. However, PFS significantly longer in the axitinib group (3.6 months) than in the placebo arm (1.8 months) in Asia (HR 0.556, 95% CI 0.370–0.835; P= 0.0023) but not in non-Asia group (P>0.05). In line with the above findings, the adverse events were hypertension, diarrhea, asthenia, and fatigue, etc. This study revealed that the PFS in the axitinib/BSC arm could be affected by different population, showing that longer PFS was identified in patients from Asia rather than non-Asia. In a phase II randomized trial developed in China, Lin et al. (49) reported the disease control rate was 62.2% in 45 advanced HCC patients received 5 mg axitinib twice daily orally. Median PFS and OS of the participants were 2.2 months and 10.1 months, respectively. Of note, the side-effects were detected as fatigue (60%), anorexia (57%), hypertension (56%), and rash (49%), which were higher than that of other phase II studies. This study observed that Axitinib is moderately active and has acceptable toxicity for patients with advanced HCC who have failed to respond to sorafenib monotherapy in the first instance.

In addition to the above clinical trials, Zhang et al. (16) presented a case report on the topic of the combination of axitinib and cabozantinib after failed sorafenib treatment in a metastatic HCC patient. This male patient aged at 64 y old, complained of discomfort in upper abdomen. This patient was diagnosed with lung and bone metastases of stage D primary HCC with chronic type B hepatitis. The author reported that this advanced HCC patient received the combination of axitinib (7 mg twice daily orally) and cabozantinib (50 mg, qd, orally) after failed sorafenib treatment. However, the patient had a total survival of 10 months after these treatments. The patient exhibited disease progression after treating with sorafenib for 2.5 months. Then, he was treated with treated with angiogenesis inhibitor axitinib and c-Met inhibitor cabozantinib, but turned to be a poor outcome. This study highlighted that options of appropriate therapies and timing needed enhanced communication and collaboration of relevant disciplines, which might facilitate to improve the therapeutic efficacy.

## Directions for future research

Most of the above included studies were designed for treating the advanced HCC patients with axitinib alone. The outcomes turned out to be no significant benefit from axitinib treatment on the OS. Even though this agent could prolong the OS of the sufferers, there was no significant difference between axitinib treatment and the placebo. However, we should note that patients received axitinib treatment had a significant better PFS as compared to the controls. Therefore, many scholars believed that Axitinib might serve as the second-line treatment for patients with advanced HCC who failed sorafenib treatment.

Mounting evidence demonstrated that the combination of immunotherapy (included the combination of different immunosuppressors) and radiotherapy/chemotherapy/ablation may significantly promote the therapeutic efficacy in patients with unresectable or metastatic HCC (50–53). Combination therapy may have better antitumor properties than monotherapy. Though monotherapy with axitinib showed no significant survival benefits, the combination of anti-PD-1/PD-L1, anti-CTLA-4, or other tyrosine kinase inhibitors (TKIs) may be a promising regimen for advanced HCC. Immunotherapy is a promising therapy option for unresectable and advanced HCC. Immune checkpoint inhibitors (ICIs) have been shown to be highly effective in the treatment for this type of cancer. PD-1/PD-L1 is one of the widespread applications of ICI, having the potent anticancer effect for advanced HCC. According to the lately evidence, ICIs combined with kinase inhibitors exhibit a potential superior anti-tumor effect on advanced HCC. Mechanistically, PD-1/PD-L1 blockade with its antibody can restore T-cell function, while axitinib is an inhibitor against multiple types of VEGFR. Both anti-PD-1 and anti-VEGFR enhance the therapeutic anti-tumor effects. Current evidence indicates that single-agent axitinib showed none of significant overall survival benefit for patients with advanced HCC. However, promisingly, axitinib combined with anti-PD-1/PDL-1 agents have exerted a potential anticancer effect on advanced HCC. Within the topic of this review, Kudo et al. (54) earlier detected axitinib plus anti-PD-1/PDL-1 agents had the possible antitumor potential effect on advanced HCC. In this study, axitinib combined with anti-PD-L1 monoclonal antibody (avelumab) presented with a manageable toxicity profile. 72.7% of the advanced HCC patients complicated with Grade 3 treatment-related adverse events, including hypertension, palmar-plantar erythron dysesthesia syndrome, and loss of appetite. However, no Grade 4 treatment-related adverse events or treatment-related deaths occurred under these regimens. Though the objective response rate appeared to be numerically lower compared to other similar trials in the first-line HCC setting, a combination treatment of axitinib and avelumab exerted with clinical activity as first-line treatment. The authors suggested that the inconsistent results might be correlated to the limited patient numbers and differences in trial design. Several undergoing phase 1 and phase 3 studies (55–57) have investigated the anti-PD-1 monoclonal antibody plus anti-VEGF multikinase inhibitor in patients with advanced HCC. The objective response rates were up to 36% per RECIST and 46% per mRECIST, indicating the promising anti-tumor effects on advanced HCC. Furthermore, Kudo et al. (54) also found that patients without baseline vascular invasion or with baseline extrahepatic spread presented with a higher ORR per RECIST 1.1 and favorable OS. In addition, patients with PD-L1+ tumors had longer OS than those with PD-L1- tumors. Therefore, anti-VEGF multikinase inhibitor plus anti-PD-1 monoclonal antibody may be more suitable for the patients who without baseline vascular invasion, with baseline extrahepatic spread, or with PD-L1+ tumors. The United States FDA approved sorafenib and nivolumab as the first anti-PDL-1/PD-1 antibodies for the treatment of HCC. Since both sorafenib and axitinib are the important tyrosine kinase inhibitors, axitinib combined with anti-PDL-1/PD-1 antibodies may also have promising outcomes in advanced HCC treatment, which is waiting for more well-designed RCT to prove it.

At present, there were two phase I trials have been published recently within the topic of the combination of axitinib and other therapies. Both of the two studies were conducted in Asia (China and Japan) and published in the year of 2021. Kudo et al. (54) recruited 22 advanced HCC patients who aged 20-84 in a phase I study. The authors found that advanced HCC patients received 5 mg axitinib twice daily orally combined avelumab 10 mg/kg intravenously every 2 weeks had a moderate objective response rate (13.6%, 95% CI: 2.9–34.9%, per RECIST 1.1 and 31.8%, 95% CI: 13.9–54.9%, per mRECIST). The adverse events included hypertension (50.0%), palmar-plantarerythrodysesthesia syndrome (22.7%), and decreased appetite (13.6%). In another recent phase I trial, Yang et al. (58) demonstrated that nine advanced HCC patients under 1 mg, 2mg, and 3mg axitinib twice daily for 8 weeks in combination with radiotherapy had an overall response rate of 66.7%. The 1−year OS was recorded at 66.7% and median PFS was 7.4 months. The side-effects included hypertension, proteinuria, increased alanine transaminase, alkaline phosphatase, and bilirubin. The two phase I study suggested that axitinib in combination with other therapies exhibited a promising antitumor efficacy on advanced HCC and underwent with a comparable adverse events of axitinib monotherapy. However, these results were derived from phase I or phase Ib trial with a small sample size. Therefore, more multi-center randomized trials with large sample size are still warranted for validating the therapeutic efficacy of the combination of axitinib and other treatments in managing patients with advanced HCC.

## Conclusion

The present review highlights the current clinical applications and the molecular mechanisms of axitinib in advanced HCC. The included randomized or single-arm phase II trials indicated that axitinib could not prolong OS as compared to placebo for the treatment of advanced HCC, but improvements in PFS and time to tumor progression were observed. Experimental studies showed that the biochemical effects of axitinib in HCC might be regulated by its associated genes and affected signaling cascades. Axitinib combined with axitinib exerts promising antitumor efficacy on advanced HCC. Future directions should focus on the identification of precise biomarkers and the development of novel immunotherapy agents. To move toward clinical applications by combining axitinib and other treatments in advanced HCC, more studies are still warranted in the near future.

## Author contributions

HJ and SX contributed to conceive and design the study. JL, CJ, and JM performed the article searching. HJ and SX extracted the data. HJ and SX wrote the manuscript. JM and LW supervised the manuscript. All of the authors read and approved the final manuscript.
